# Notch Signaling Regulates Immune Responses in Atherosclerosis

**DOI:** 10.3389/fimmu.2019.01130

**Published:** 2019-05-22

**Authors:** Francesco Vieceli Dalla Sega, Francesca Fortini, Giorgio Aquila, Gianluca Campo, Mauro Vaccarezza, Paola Rizzo

**Affiliations:** ^1^Translational Research Center, Maria Cecilia Hospital GVM Care & Research, Cotignola, Italy; ^2^Department of Medical Sciences, University of Ferrara, Ferrara, Italy; ^3^Cardiovascular Center, Azienda Ospedaliero-Universitaria di Ferrara, Cona, Italy; ^4^Faculty of Health Sciences, School of Pharmacy and Biomedical Sciences, Curtin University, Perth, WA, Australia; ^5^Department of Morphology, Surgery, and Experimental Medicine, University of Ferrara, Ferrara, Italy; ^6^Laboratory for Technologies of Advanced Therapies, University of Ferrara, Ferrara, Italy

**Keywords:** atherosclerosis, endothelial dysfunction, Notch, Dll4, immunity, M1 macrophages, T cells, GSI

## Abstract

Atherosclerosis is a chronic autoimmune inflammatory disease that can cause coronary artery disease, stroke, peripheral artery disease, depending on which arteries are affected. At the beginning of atherosclerosis plasma lipoproteins accumulate in the sub-endothelial space. In response, monocytes migrate from the circulation through the endothelium into the intima where they differentiate into macrophages. These early events trigger a complex immune response that eventually involves many cellular subtypes of both innate and adaptive immunity. The Notch signaling pathway is an evolutionary conserved cell signaling system that mediates cell-to-cell communication. Recent studies have revealed that Notch modulate atherosclerosis by controlling macrophages polarization into M1 or M2 subtypes. Furthermore, it is known that Notch signaling controls differentiation and activity of T-helper and cytotoxic T-cells in inflammatory diseases. In this review, we will discuss the role of Notch in modulating immunity in the context of atherosclerosis and whether targeting Notch may represent a therapeutic strategy.

## Introduction

Atherosclerosis is widely recognized as the most common cause of coronary artery disease, periphery artery disease, and stroke and thus the most relevant player in mortality and morbidity in the entire world (http://www.who.int/healthinfo/global_burden_disease/estimates/en). The disease typically progresses with a slow build-up of lipid-laden plaques in large and medium arteries. It can remain silent for decades until plaque rupture or erosion leads to the formation of thrombus that blocks the arterial vessels leading to ischemic injury in the surrounding tissues. Atherosclerosis is deeply intertwined to chronic inflammation and immune cells are involved in all the stages of the disease (1). However, only recently, the CANTOS study provided definitive proof that targeting inflammation improves clinical outcomes in patients with atherosclerosis (2–4). After the success of the treatment with an anti-IL-1β antibody, efforts to target the immune system to counteract atherosclerosis are on the rise and potential new strategies to target immunity and inflammation are being envisioned (5). Notably, very recent data underscored the early inflammatory component of the disease in the vascular wall, with substantial arterial inflammation highly prevalent even in middle aged subjects with subclinical manifestations identified through diagnostic procedures (6, 7).

The role of Notch pathway in regulating the differentiation of the different cells of innate and adaptive immune system cells is well-recognized (8). On the other hand, small molecules Notch inhibitors and specific antibodies blocking Notch ligands or receptors have provided greats tool to gain an understanding of the role of Notch in the immune system under physiological or pathological conditions (9). The Notch pathway is a major regulator of cell fate in stem cells, thus in continuously renewing tissues and its dysregulation has been observed in pathologic states, including cancer (10). Small molecules inhibiting γ-secretase (GSI, γ-secretase inhibitors), the enzyme necessary for the activation of Notch, have been originally developed because γ-secretase is also involved in a β-amyloid polypeptide, found in brains of Alzheimer's disease patients (11). During these past 20 years GSIs with different specificity and modality of action and other Notch blocking agents have been developed and are currently being investigated in clinical trials for those cancers driven by dysregulation of this pathway (12, 13).

In this Review, we will describe the role of Notch in the modulation of immune responses during the different phases of atherosclerosis and discuss available evidence suggesting that the targeting of this pathway may represent a novel therapeutic strategy for this disease.

## The Core Notch Pathway

The Notch pathway is a cell signaling system that mediates cell-to-cell communication. Notch signaling controls cell fate choices and modulates crucial cellular functions, such as cell differentiation proliferation and apoptosis (14). Mammals express four isoforms of Notch receptors (Notch 1–4), and five Notch ligands [Delta-like ligand (Dll 1, 3, and 4), and Jagged-1 and 2] (15). Notch receptors are initially synthesized as a single precursor that migrates to the Golgi apparatus where it is split by a furin-like protease into an extracellular and a transmembrane subunit (16). For the assemblage of the functional receptor the two subunits are transported to the cell-membrane where they are held together by non-covalent bonds. The binding of a Notch ligand with its receptor triggers the removal of the extracellular portion, followed by two subsequent proteolytic cuts, the first by a disintegrin and metalloprotease (ADAM10 and/or 17) and the second by a γ-secretase, a multiprotein complex membrane protease, resulting in the release of the active form intracellular Notch (NICD). In the Notch “canonical” signaling the NICD migrates into the nucleus where it controls the transcription of target genes through binding to transcription factors recombinant binding protein for the immunoglobulin region κJ (RBPJ) mediating the displacement of co-repressors and the recruitment of Mastermind proteins (MAML 1–3). The NICD/RBPJ/MAML complex recruits additional co-activators, such as p300 and PCAF to guide the transcriptional expression of the genes under the control of Notch. The best characterized Notch target genes belong to Hes (Hairy and Enhancer of Split) and Hey (Hairy and Enhancer of Split with YRPW) families of transcriptional repressors (17). The Notch signaling can also function through “non-canonical” pathways: the NICD activity can be independent of RBPJ; the activation of γ-secretase can initiate the Notch pathway independently of the binding with canonical ligand; or Notch signaling is activated in the absence of the cleavage of the γ-secretase complex (18). Non-canonical Notch signaling involves interactions with Wnt/β-catenin (19), mTORC2 (mammalian target of rapamycin complex 2)/Akt (20), or IKKα/β (18) pathways, and can also occur in mitochondria where Notch/PINK1 (PTEN-induced kinase 1) complexes modulate mitochondrial metabolism promoting cell survival by activating the mTORC2/Akt pathway (21).

## The Onset of Atherosclerosis Involves Endothelial Dysfunction: the Role of Notch

At the onset of atherosclerosis plasma lipoproteins accumulate in the subendothelial space triggering inflammatory responses that eventually result in the expression of adhesion molecules and in the impairment of endothelial physiology. Various lines of evidence have shown that Notch signaling in ECs is dysregulated by atherogenic stimuli, such as inflammatory cytokines (22–24), dyslipidemia (25, 26), and disturbed shear stress (27–29). Conversely, it is well-established that a functional Notch signaling provides protection from endothelial dysfunction induced by various atherogenic stressors (23, 24, 29).

Atherosclerotic lesions arise primarily in specific areas of the arteries, such as bifurcations or curvatures characterized by a turbulent blood flow and a disturbed shear stress. In recent times, data supporting a role for Notch as a transducer of laminar shear stress have been emerging (28). It has been reported that low shear stress induces the downregulation of miRNA126-5p resulting in the upregulation of Dlk1 which, by inhibiting Notch1, hinders ECs proliferation required for the efficient renewal of the endothelium damaged by dyslipidemia. Importantly, Mir126(–/–) mice exhibited exacerbated atherosclerosis (30). In agreement with this study, a reduced expression of the Notch signaling components was found in the atheroprone regions of mouse aorta (27). Moreover, in ApoE-deficient mice the modulation of shear stress with ivabradine, a heart rate reducing drug, protected the endothelium in the first phase of atherosclerosis through a mechanism depending on Notch signaling (31). Molecular mechanisms underlying the anti-atherogenetic effects of laminar shear stress through Notch have been recently described by Mack et al. (29). In this study, the lack of endothelial Notch1 results in hypercholesterolemia-induced atherosclerotic lesions in the descending aorta. Additionally, the authors have shown that, in the endothelium, Notch1 is activated by shear stress and that it is necessary for the maintenance of junctional integrity. Conversely, the reduction of Notch1 weakens endothelial junctions and causes ECs proliferation. A role of Notch in the transduction of shear stress has also been observed by Polacheck et al. that demonstrated that shear stress-induced Notch1 activation is crucial for maintaining the endothelial barrier function. In this work, findings from experiments *in vivo* and in an organotypic model of microvessels that could be perfused at different shear stress, revealed that Notch1 controls vascular barrier integrity through non-canonical signaling: shear stress triggers a Dll4-dependent proteolytic activation of Notch1 that determines transmembrane domain (TMD) exposure which is required for the assembly of the endothelial junction complex (32). Recently, Miyagawa et al. showed that contacts between ECs and SMCs are necessary for the activation of Notch1 mediated by BMPR2 (bone morphogenetic protein receptor 2). In ECs, BMPR2 drives the translocation of p-JNK (phospho-c-Jun N-terminal kinase) to the cell membrane stabilizing presenilin1 and activating Notch1. Notch1 promotes ECs proliferation sustaining glucose metabolism and mitochondrial activity, and it is required for the integrity of endothelium and for its regeneration following an injury (33).

An early study in cultured cells by Quillard et al. (34) showed that TNF-α impairs Notch signaling by altering Notch4 and Notch2 levels; in turn, the dysregulation of Notch pathway promotes apoptosis through the downregulation of the anti-apoptotic protein survivin (23). Interestingly, in the Quillard studies, the Notch alteration was linked with an induction of the VCAM-1 and ICAM-1 adhesion molecules. The discovery that Notch signaling downregulates the expression of adhesion molecules was subsequently confirmed and extended by Briot et al. which demonstrated that Notch signaling in the endothelium is curbed by various pro-atherogenic stimuli and that Notch1 is essential to impede the expression of inflammatory molecules and the binding of monocytes (25). In this study Notch1 was found down-regulated in aortic ECs in response to a high-fat diet or to exposure to pro-atherogenic oxidized lipids or inflammatory mediators TNF-α and interleukin-1β (IL-1β). Decreased Notch1 signaling promoted inflammatory cell binding to ECs and increased expression of pro-inflammatory molecules IL-8 and CXCL1. Of note, Notch antagonized inflammatory phenotype when the protein was ectopically overexpressed in ECs exposed to stressors that cause Notch suppression (25).

Emerging evidence shows that Notch signaling mediates communication between EC and immune cells after endothelial activation induced by atherogenic stress factors. Pabois et al. have shown that TNF-α drives the endothelial expression of Dll4 which, in turn, promotes the polarization of macrophages to a pro-inflammatory phenotype that induces IL-6 production (35). Moreover, it was recently found that, in mice, endothelial Dll1 drives the Notch2 dependent conversion of Ly6C(hi) (inflammatory) monocytes into Ly6C(lo) (patrolling) monocytes (36). Furthermore, Krishnasamy et al. have recently reported that macrophage maturation is controlled by Dll1 expressed in ECs and requires the canonical signaling of RBPJ in macrophages, which simultaneously suppresses an inflammatory polarization of macrophages. Conversely, mice lacking Dll1 or RBPJ showed an accumulation of inflammatory macrophages resulting in compromised tissue repair and arteriogenesis (37). Interplay between ECs and macrophages has been also shown *in vitro* co-cultures: specifically sprouting angiogenesis is enhanced in co-culture of ECs with M1 polarized macrophages, but not with M2 activated macrophages, and this effect is dependent on Notch signaling (38).

## Notch Regulates Macrophages-Mediated Inflammation in Atherosclerosis and Ischemic Heart Disease

In the early stages of atherosclerosis circulating monocytes bind to ECs expressing adhesion proteins and migrate to the intima where they differentiate into macrophages. During the progression of atherosclerosis, monocytes attracted by inflammatory cytokines continue to infiltrate the growing plaque contributing to perpetuate the inflammation. Macrophages are classically divided into a high-inflammatory M1 subset and an anti-inflammatory (or less-inflammatory) M2 subset. M1 macrophages are classically defined as pro-inflammatory players secreting cytokines, such as IL-1, IL-6, IL-12, IL-15, IL-18, MIF, TNF-α able to trigger T cell-mediated responses. M2 macrophages hold anti-inflammatory activities able to resolve plaque inflammation and release different cytokines (IL-4, IL-10, and IL-13) from M1 (39). TGF-β produced by M2 macrophages has a role in the biology of the vascular wall by influencing cell proliferation, differentiation, and production of extracellular matrix (40). Overall, inflammatory macrophages (M1) sustain mechanisms that favor atherosclerosis progression, whereas M2 macrophages drive mechanisms that are able to suppress plaque formation and progression and even to support plaque regression (39). Interestingly, the number of M1 and M2 macrophages changes depends on the plaque field. For example, M1 macrophages are abundant in regions that are inclined to rupture. On the contrary, M2 macrophages are more abundant in areas where thicker fibrous caps and smaller areas of necrosis are present, demonstrating the plaque–stabilizing function of macrophages (41, 42). A comprehensive discussion of macrophages' role can be found in recent reviews (5, 39).

Studies on cultured monocytes found that Notch1 induces M1 macrophage differentiation and heightens inflammatory responses by increasing IL-6, MCP-1, and TNF-α production. Conversely, Notch1 inhibition drives in the direction of an increase of M2 differentiation promoting the secretion of anti-inflammatory cytokines IL-10 and IL-1RA (43, 44). Aoyama et al. have shown that in ApoE^−/−^ mice, the treatment with Notch inhibitor DAPT reduced macrophages migratory activity and repressed ICAM-1 expression in macrophages that led to decreased macrophage infiltration in the atherosclerotic plaques (45). The first direct evidence of Notch involvement in regulating functions of human macrophages in atherosclerosis stems from a study by Fung et al. in which the authors observed the expression of Dll4 and Notch3 in infiltrating macrophages and atherosclerotic plaques. In this study, *in vitro* experiments with pro-inflammatory molecules, such as LPS, IL-1β, or modified LDL have been shown to promote the expression of Dll4 in macrophages. Dll4, in turn, causes additional pro-inflammatory responses in a manner dependent on Notch receptors thereby triggering a positive feedback loop in plaque macrophages (46). Pabois et al. have shown that, during microvascular inflammation, there is an increase in the expression of Dll4 in both ECs and macrophages, suggesting that Dll4 may be a marker of endothelial activation and could play a role in endothelial/macrophage interactions during inflammation (35). Recently, the same group demonstrated that Dll4 is the ligand involved in the Notch-dependent selection process promoting the differentiation of M1 macrophages and preventing the differentiation of M2 macrophages blocking the expression of M2 genes induced by IL-4. Noteworthy, Dll4 was also able to promote the induction of apoptosis selectively in M2 cells (47). Consistent with a pro-inflammatory role of Notch signaling, Fukuda et al. have been shown in LDLr^−/−^ mice that high-fat/high-cholesterol diet promotes expression of Dll4 in the atherosclerotic plaques and in fat tissue. Inhibition of the Notch signaling with anti-Dll4 antibody reduced atherosclerotic lesions, diminished plaque calcification while improving insulin resistance, and decreasing fat accumulation. These changes were associated with a reduction of macrophage accumulation and decreased MCP-1 levels. *In vitro* experiments revealed that Dll4-mediated Notch signaling increases MCP-1 expression by activating NF-κB. Noteworthy, also in this setting Dll4 induced macrophages M1 polarization (48). Nakano et al. reported that in a mice model of chronic kidney disease (CKD) accumulation of the uremic toxin 3-indoxylsulfate drives the expression of Dll4 in macrophages with consequent Notch signaling-induced pro-inflammatory responses. In this model an anti-Dll4 antibody was able to lessen both macrophage accumulation and atherosclerosis (49). More recently, it has been shown that the inhibition of Furin, an enzyme involved in Notch1 activation (16), reduces atherosclerosis progression in LDLr^−/−^ mice. Of note, mice treated with furin inhibitors also had reduced inflammation and less macrophages in the plaque (50).

Molecular details of the function of Notch in activated macrophages have been investigated in cultured cells. *In vitro* experiments found that Notch1 activation occurs in response to LPS or IFN-γ and is implicated in macrophage activation by upregulating the expression of ICAM-1 and major histocompatibility class II antigens (MHCII) in macrophages (51). In addition, it has been shown that Notch1 positively regulates IL-6 production via NF-κB in activated macrophage (52). Following binding to its receptor, IL-6 activates STAT3 that, in turn, induces Dll1 expression that initiates the Notch signaling. Notch increases NF-κB activation that results in IL-6 production which transduces stabilization of STAT3 activation establishing a positive feedback loop (53). Notch-RBPJ signaling regulates the transcription factor IRF8 to promote inflammatory macrophage polarization and expression of prototypical M1 effector molecules, such as IL-12 and iNOS (54). Conversely, blockade of canonical Notch signaling was shown to decrease macrophage-mediated inflammation correlating with improved late wound healing in diabetes (55). Recently, the role of Notch in regulating M1/M2 polarization was confirmed by Huang et al., that found that miR-148-3p is expressed following activation of Notch1 and promotes M1 polarization while inhibiting M2 differentiation (56). Furthermore, it has been reported that in ApoE^−/−^ mice the anti-atherosclerotic miR-181b modulates macrophage polarization by directly targeting Notch1 (57). Importantly, Xu et al. showed that Notch1 induces an increase of mitochondrial glucose oxidation that in turn triggers the expression of M1 pro-inflammatory genes (58).

Apparently in contrast with previous studies, Fondi et al. have shown that Notch is involved in polarization of M2 macrophages. In this study, it was seen that mice deficient in RBPJ in myeloid cells poorly differentiate into M2 (59). In addition, Onishi et al. have shown that Dll1 inhibits GM-CSF-dependent differentiation of monocytes into mature macrophages but promotes differentiation of dendritic cells (DCs) progenitors and further differentiation into mature DCs in the presence of GM-CSF, IL-4, and TNF-α (60).

It is well-established that M1 macrophages infiltration worsen ischemic damage after myocardial infarction (MI) hindering the resolution of inflammation and scar formation; by contrast, the presence of anti-inflammatory M2 macrophages in the infarct area facilitate pro-reparative processes (61). Yin et al. have shown in a rat model that after MI, M1 macrophages that infiltrate the infarct area express high levels of Notch1. The administration of the Notch inhibitor DAPT 30 min prior to MI caused a decrease of total macrophages in the infarct area, but enhanced the ratio of M2-activated macrophages. Furthermore, rats pre-treated with DAPT had a decrease in the cardiac re-innervation after MI, this eventually resulted in a better recovery of heart electric functionality after MI (62). The expression of the C-C chemokine receptor type 2 (CCR2) in macrophages is controlled by RPBJ (63). Recently, Bajpai et al., found that, following MI, tissue resident CCR2^+^ macrophages promote the recruitment of inflammatory monocytes to the injured heart. These monocytes secrete pro-inflammatory cytokines contributing to the adverse cardiac remodeling. On the contrary, resident CCR2-macrophages inhibit pro-inflammatory leukocyte recruitment protecting from adverse remodeling after MI (64, 65).

Overall, these findings indicate that Notch signaling in monocytes and vascular macrophages promotes inflammation by facilitating a pro-inflammatory M1 phenotype at the expense of the anti-inflammatory M2 subtype. In this process, the axis Dll1-Dll4/Notch1 plays a crucial role both by initiating M1 program and inhibiting M2 differentiation.

## Functional Phenotypes of T-Cells Determine Atherosclerosis Progression: a Possible Role of Notch

In T cells activation, the MHC molecules interact with oxLDL, microbial antigens, and heat shock proteins (HSP 60), which help to protect cells from stress damage driven by stressed endothelial cells. Furthermore, engagement of the co-stimulatory molecule CD28 to T cells allows interactions with CD80 or CD86 on antigen-presenting cells (APCs). As for monocytes/macrophages, T cell functional phenotypes can be modified by environmental factors and different “pabulum,” thus modulating their possibility to act as regulatory or inflammatory cells.

The importance of Notch signaling in T cells has been established in diseases of autoimmune and inflammatory origin, but studies directly addressing the role of Notch in atherosclerosis are lacking. In this section, we will describe how Notch regulates the functionality of T cells in immune/inflammatory diseases and the putative role of Notch in modulating adaptive cells in the progression of atherosclerosis.

### Notch in T-Helper Cells

Most of the T cells present in human plaques are CD4 T-helper (Th) cells and different T-helper cell subgroups arise following micro-environment cues and following encounter with APCs. Th1 cells secrete IFN-γ, IL-2, IL3, and TNF-α and have been shown to be the main subtype in human atherosclerotic plaques and the pro-atherosclerotic effect of these cells have been shown in several animal studies (1). IFN-γ is a pro-atherogenic cytokine and growth inhibitor of SMCs and ECs that also affects macrophage polarization. After arterial damage, growth of SMCs is inhibited by IFN-γ secreted from Th1 cells, which determines atherosclerotic plaque destabilization and rupture. Furthermore, IFN-γ increases TNF-α and IL-1 production, which are strong pro-inflammatory molecules and indirectly inhibit the proliferation of SMCs and endothelial cells (66).

The capacity of Notch ligands Dll1/Dll4 to promote Th1 cell differentiation is supported by many *in vitro* and *in vivo* studies in which Notch inhibition was achieved by different approaches. Maekawa et al. have shown in cultured T cell that soluble Dll1 induces T-cells differentiation into IFN-γ secreting Th1 phenotype (67). In DCs/T-cells co-culture it has been shown that Dll4-deficient DCs have limited capacity to induce CD4 T-cell activation, proliferation, and cytokines secretion (68). In mice, treatment with γ-secretase inhibitors reduced disease progression in a Th1 cell-mediated experimental autoimmune encephalomyelitis (EAE) (69) while the deletion of Dll4 from DCs resulted in a reduced ability to mount a CD4-dependent response in mice (68). Furthermore, Riella et al. have shown that Dll1 blockade results in a Th1 decrease in an allograft model (70). Interestingly, anti-Dll4 antibodies diminished T-cells secretion of IFN-γ and TNF-α (71) suggesting that Delta-ligands can not only affect differentiation but also regulate cytokines secretion in differentiated Th1 cells. Studies in transgenic mice unable to activate RBPJ because of dominant-negative MAML expression, showed that canonical Notch signaling is not involved in Th1 polarization (72), and similarly, in T-cells lacking RBPJ expression, the capacity to drive a Th1 cells in response to infection was maintained (73). Dongre et al. confirmed that differentiation to Th1 cells occurs independently from RBPJ and demonstrated that Notch signaling triggers Th1 polarization by non-canonical signaling involving Notch1-dependent activation of NFκB pathway (74).

Th17 cells are characterized by the expression of RORγt and the production of IL-17 which have been linked to the atherosclerosis (75). Under the effect of inflammatory cytokines, Th17 cells can be switched from barrier-protective IL-10 secreting T-effector cells (T-eff) into pathogenic drivers that produce IL-22, and IFN-γ (76). IL-1, IL-6, and IL-23 drive this Th17 switch into pathogenic effector cells. Of note, dual IL-17/IFN-γ-producing Th17 cells are present in atherosclerotic human coronary arteries at a higher frequency than in the general circulation (77). In the last decade, accumulating evidence has highlighted the role of Notch in regulating differentiation and functionality of the Th17 subset. It is well-established that, in EAE, Th17 cells arise from naïve T-cells in the central nervous system where, along with Th1, promote autoimmunity (78). In this context, it has been shown that Notch inhibition, by γ-secretase inhibitors (69, 71) or by antibodies against Dll1 (79), results in a decrease of Th1 and Th17 cells. Noteworthy, DCs expressing high levels of Dll4 have greater ability than other DCs to promote the generation of Th1 and Th17 from naïve T-cells (80, 81). Conversely, blocking Dll4 with antibodies decreased Notch signaling in T cells stimulated with Dll4 expressing DCs, thus reducing Th1 and Th17 cells (82). Administration of DAPT repressed Th1- and Th17-mediated responses in spleen and lymph nodes resulting in a decrease of circulating IFN-γ and IL-17 in a mouse model of arthritis (83). Meyer Zu Horste et al. have shown in transgenic mice that RBPJ deletion in T-cells did not impair Th17 differentiation induced by TGF-β1 and IL-6. Nevertheless, in the same study, it was seen that RBPJ determines the pathogenicity of Th17 cells by regulating IL-23R and IL-10 expression (84). These findings indicate that canonical Notch signaling may modulate differentiation and cytokines secretion. Contrary to the effect observed for Delta-like- ligands, Jagged-1 suppressed Th17 cell differentiation induced by IL-6 and TGF-β (85). Recently, Zaman et al. found that DCs lacking RBPJ promote the generation of Th17 cells but have limited capacity in instructing CD4 cells to differentiate into Tregs in the presence of TFG-β, thus suggesting that Notch signaling in DCs has a crucial role in determining balance between Th17/Tregs (86).

Th9 cells are characterized by production of IL-9, their development occurs from naive CD4 T cells in the presence of IL4 and TGF-β (87). Some evidence suggests that IL-9 producing Th9 cells help the development of atherosclerosis (88). Elyaman et al. have reported that Jagged2, but not Dll1, induces Th9 cell polarization. Moreover, CD4 T cells lacking Notch1 and Notch2 display a reduced capacity to differentiate into Th9 when exposed to IL-4 and TGF-β (89).

Th2 cells are present in atherosclerotic plaques at lower frequency compared to Th1 cells. Initially, Th2 cells have been thought to act as anti-atherosclerotic by opposing Th1 phenotype (90) but subsequent studies lack clear demonstration of the atheroprotective role of Th2 cells. Engelbertsen (91) et al. reported that subjects with a high ratio of circulating Th2 cells has a lower cardiovascular disease risk, but triggering a Th2 response did not exert an anti-atherosclerotic effect in ApoE^−/−^ mice. Zhao et al. found that IL-4 (the main driver of Th2 differentiation) protects against atherosclerosis by promoting M2 over M1 macrophage inflammatory phenotype (92). By contrast, IL-4 delivery was not able to decrease atherosclerosis in murine models of atherosclerosis (93, 94).

Different lines of evidence have shown that canonical Notch signaling instructs for Th2-type response. CD4 T cells lacking RBPJ retain limited capacity to differentiate into IL-4 producing Th2-type while cells CD4 T cells deficient for Notch-1 and Notch-2 completely lose the capacity to mount a Th2-type response (95). RBPJ binding sites presence on IL-4 gene enhancers regions suggests a direct role of Notch in IL-4 transcription in Th2 cells and possibly in other IL-4-secreting cells (95, 96). The capacity of Notch to control Th2 cell differentiation has been shown in many experimental models *in vivo* (96, 97). The production of specific cytokines in Th2 cells is under control of the transcription factor Gata3 (98). Notch1 and RBPJ both bind in the proximity of Gata3 promoter and the activity of this complex is required for the Notch-dependent production of IL-4, indicating that Notch controls Th2 cells differentiation and IL-4 production by directly promoting Gata3 expression (97, 99). In mice, expression of Jagged2 in DCs promotes Th2 cells polarization and IL-4 production by activating Notch1 canonical signaling (100). Tindemans et al. have shown in a murine model of asthma that RPJB in T-cells is necessary for the induction of a Th2-mediated allergic inflammation but, in this model, Jagged1 or Jagged2 deletion on DCs did not affect the capacity to promote a Th2 immune response (101). This may indicate that Notch signaling could be activated to induce Th2 cells also through ligands-independent pathways. Interestingly, Damle et al. found that mice with DCs lacking ADAM10 are unable to produce the cytokines necessary for Th2 differentiation but maintain the ability to drive Th1 and Th17 immunity (102). In this study, the reactivation of Notch signaling with N1ICD overexpression rescued DCs capacity to induce a Th2 response (102). This suggests that, in DCs, not only Notch ligands, but also Notch receptors, play a role in instructing Th-cells toward Th2 phenotype.

Regulatory T cells (Tregs) are a subpopulation of CD4 T-helper cells that modulate immunity. Regulatory T-cells hinder the differentiation of CD4 T-cells into effector subtypes and modulate APCs activity by producing modulatory cytokines, such as IL-10, IL-35, and TGF-β. It has been convincingly shown that Tregs have a protective role in atherosclerosis (103, 104). Human atherosclerosis is linked with low circulating Tregs (105), Tregs are also detectable in atherosclerotic plaques (106) where their number positively correlates with plaque stability (107). On the contrary, lower numbers of Tregs are correlated with a heightened infiltration of pro-inflammatory leukocytes into the plaque (108). Tregs depletion in ApoE^−/−^mice increased atherosclerotic lesions and plaque instability (109, 110). Several studies, *in vitro* and in animal models of inflammatory diseases, found that Notch signaling increases Tregs population (111). Notch-induced Tregs have been shown to decrease severe allergic airway inflammation (112, 113) to prolong allograft survival (114), and to alleviate the progression of autoimmune diabetes (115, 116).

Importantly, atherosclerotic and hypercholesterolemic microenvironments drive Tregs cell plasticity. CD4^+^CCR5^+^IFN-γ^+^FoxP3^+^T-bet^+^ cells (referred to as Th1-Tregs) are derived from Tregs and their presence is increased in atherosclerotic lesions of ApoE^−/−^ mice (117). Noteworthy, Th1-Treg cells display deficient regulatory functions *in vitro* and impaired expression of genes linked to Treg cells immunosuppressive activity (117). Up to 40% of the CD4 T cells in atherosclerotic aorta of ApoE^−/−^ mice showed a CCR5^+^FoxP3^+^T-bet^+^ phenotype and secreted relevant levels of IFN-γ and TNF-α (118). These cells, named FoxP3^+^CCR5^+^CD25^−^ Teff cells by the investigators, trigger atherosclerosis in adoptive-transfer experiments and do not suppress Teff cell growth. In already established Tregs, the deletion of RPJB or Notch1 increases the ability of Tregs to suppress Th1 responses, while the ectopic expression of Notch1 in Tregs causes an increase in Th1 activity (119). Similarly, in Tregs isolated from a mice model of autoimmune uveitis, it has been shown that Jagged1 and Dll1 downregulates Foxp3 expression limiting the immunosuppressive activity of Tregs. Conversely, antibodies against Jagged1 and Dll1 rescued Foxp3 levels. Importantly, transplantation of Tregs with Notch1 deficiency resulted in an increase in the release of inflammatory cytokines and in cellular infiltration in the uveitic eyes (120).

In summary, findings indicate that Notch sustains Tregs differentiation from progenitors, by contrast, in differentiated Tregs, Notch promotes a switch toward a pro-inflammatory phenotype.

### Notch in CD8 Cytotoxic T Cells

CD8 cytotoxic T cells are activated following the binding of their T-cell receptor (TCR) to the major histocompatibility complex (MHC) expressed on APCs. CD8 cells cytotoxic activity is related to the secretion of the effector proteins perforin, granulysins, and granzymes that trigger apoptosis of target cells by forming pores on their membranes (121). In addition, CD8 T cells produce TNF-α and IFN-γ that locally potentiate cytotoxic effect further fuelling inflammation (122). CD8 cytotoxic T cells are present in human atherosclerotic lesions, as proven decades ago (123), and their number is linked with atherosclerosis pathophysiology (124). In the plaque, CD8 T cells exacerbate inflammation, and exert cytotoxic activity toward lesion-stabilizing cells, such as smooth muscle cells and ECs, driving the atherosclerosis progression and plaque instability (125). By contrast, it must be noted that CD8 T-cell subsets with immunomodulatory capacity which limit atherosclerosis are also present in the plaque (125).

An early study by Wong et al. has found that Dll1 binding to splenic CD8 T cells results in a strong decrease of IFN-γ production with a concomitant increase of IL-10 production (126). The involvement of Notch pathway was also observed in peripheral CD8 T cells in which it was shown that Notch is necessary for TCR-mediated activation and that Notch inhibition blocks IFN-γ production (127). Furthermore, it has been reported that inhibition of Notch signaling in CD8 T cells blocks the production of TNF-α and cytotoxic effector molecules perforin and granzyme B (128, 129). Noteworthy, Maekawa et al. have shown that DCs expressing high levels of Dll1, trigger in CD8 T cells a higher production of cytotoxic molecules. In the same study, authors observed that Notch2-deficient T cells poorly differentiate into cytotoxic CD8 T cells (130). Activated CD8 T cells can differentiate into terminal effector cell (TEC) or can become memory precursor cell (MPC). In CD8 T cells, the concomitant Notch1 and Notch2 deficiency, or the lack of RBPJ, reduced TEC differentiation resulting in defects in host defense and eradication of tumors (131). Taken together these results suggest that Notch activates CD8 T cells at different levels. Notch signaling in CD8 T cells promotes the cytotoxic activity of the effector cells, in addition Notch instructs CD8 T cells toward TEC differentiation.

## Notch Modulation of the Crosstalk Between Innate and Acquired Immunity May Control Atherosclerosis Progression

DCs constitute a bridge between the innate and the adaptive immunity and the role of Notch signaling as mediator of communication between DCs and T-cells has been extensively investigated (132). As previously described, DCs express Notch ligands Dll1, Dll4, Jagged-1, Jagged-2, while T-cells express Notch receptors. The Notch ligand-receptor associations in DCs/Th initiate the differentiation program toward a specific T cell functional phenotype (95). For instance, it has been shown that DCs expressing Dll1 or Dll4 promote the differentiation toward pro-atherosclerotic Th1 (68, 69, 82). On the contrary, Jagged ligands instruct T cells toward the less inflammatory Th2 and Th9 subtype (132).

Myeloid-derived suppressor cells (MDSCs) constitute a heterogeneous population of immature myeloid cells that originate in the bone marrow during inflammatory diseases and migrate to inflamed tissue where they strongly suppress T-cell responses in autoimmunity (133). Recently, the murine MDSCs subset CD11b+Gr1+ has been found to possess anti-atherosclerotic activity in LDLr^−/−^ mice as MDSC adoptive transfer in these animals decreased the number and activity of Th1 and Th17 cells in the plaque and reduced the atherosclerotic lesions (134). Acute coronary syndrome (ACS) patients displayed expansion of MDSC population identified as CD14^+^HLA-DR-/low that when isolated from ACS patients MDSCs were found to contrast T-cell proliferation and IFN-γ production *in vitro* more efficiently compared to MDSC isolated from healthy or stable angina subjects (135). Transgenic mice overexpressing ADAM10, an enzyme involved in the activation of Notch, displayed a systemic expansion of CD11b^+^Gr1^+^ MDSCs (136). Conversely, blockage of Jagged1 and Jagged2 in MDSCs have been shown to inhibit T-cell repression activity of these cells (137). In light of these findings, the role of Notch in modulating the interactions between innate and adaptive immunity, may be also relevant in the progression of atherosclerosis as already seen in cancer (138). However, it must be noted that the role of Notch signaling in MDSCs has been mainly studied in the context of cancer and there is still a lot to be understood about these cells. For a comprehensive and critical discussion of the role of Notch in MDSCs we refer the reader to the following references (111, 138, 139).

## Conclusive Remarks and Clinical Perspectives

**Figure 1:**
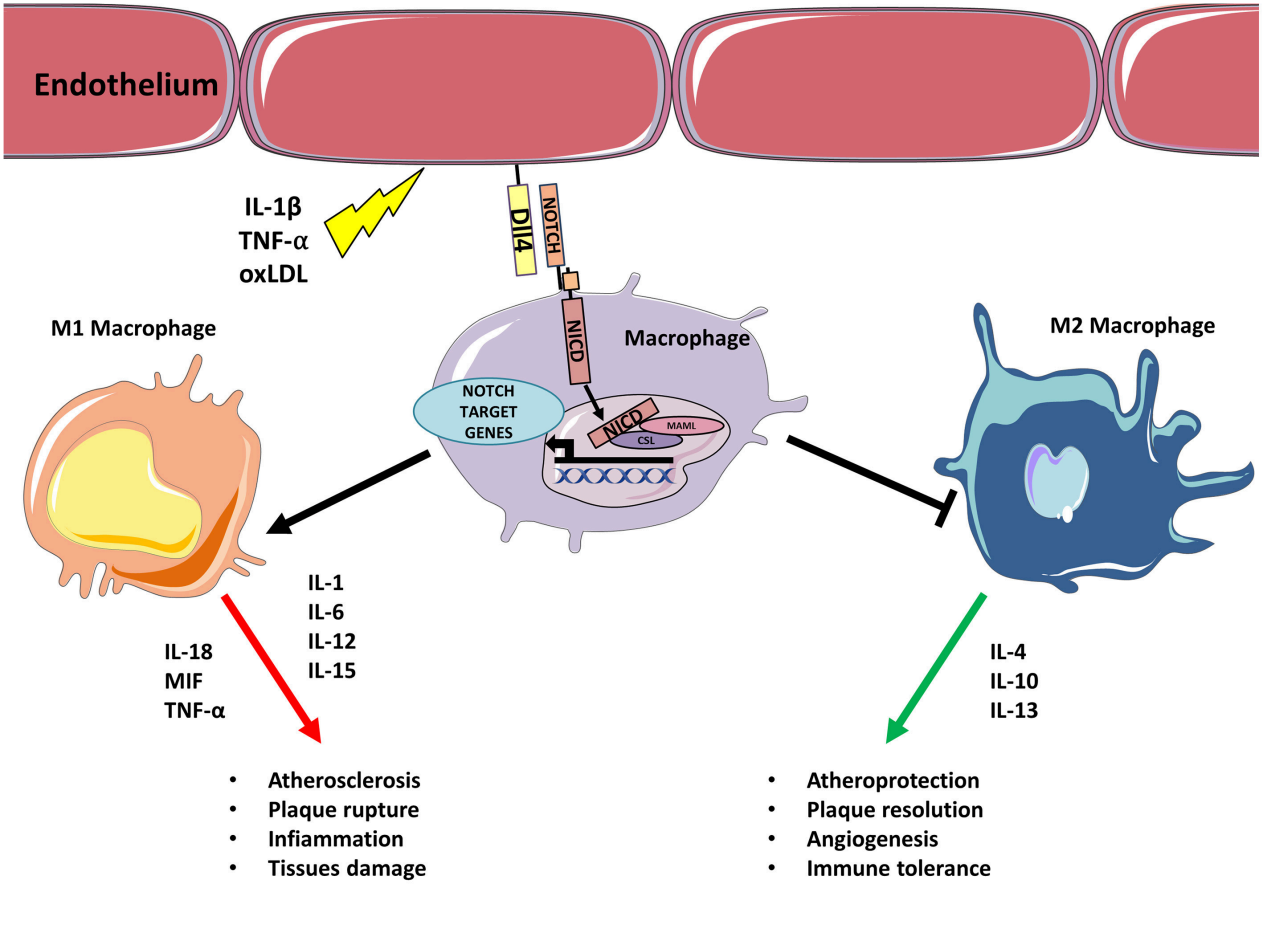
Inflammatory stimulus triggers M1 polarization via Notch. Inflammatory stimulus, such as IL-1β, TNF-α, oxLDL upregulates Dll4 on endothelial cells (or APCs). Binding between endothelial Dll4 (and possibly Dll1) and Notch1 or Notch3 in macrophages initiates Notch program that results in macrophages M1 polarization and concomitant inhibition of M2 differentiation. M1 activated macrophages feed inflammation and atherosclerosis by secreting further inflammatory cytokines, Notch blocks M2 polarization inhibiting M2 capacity to resolve inflammation/lesion.

**Figure 2:**
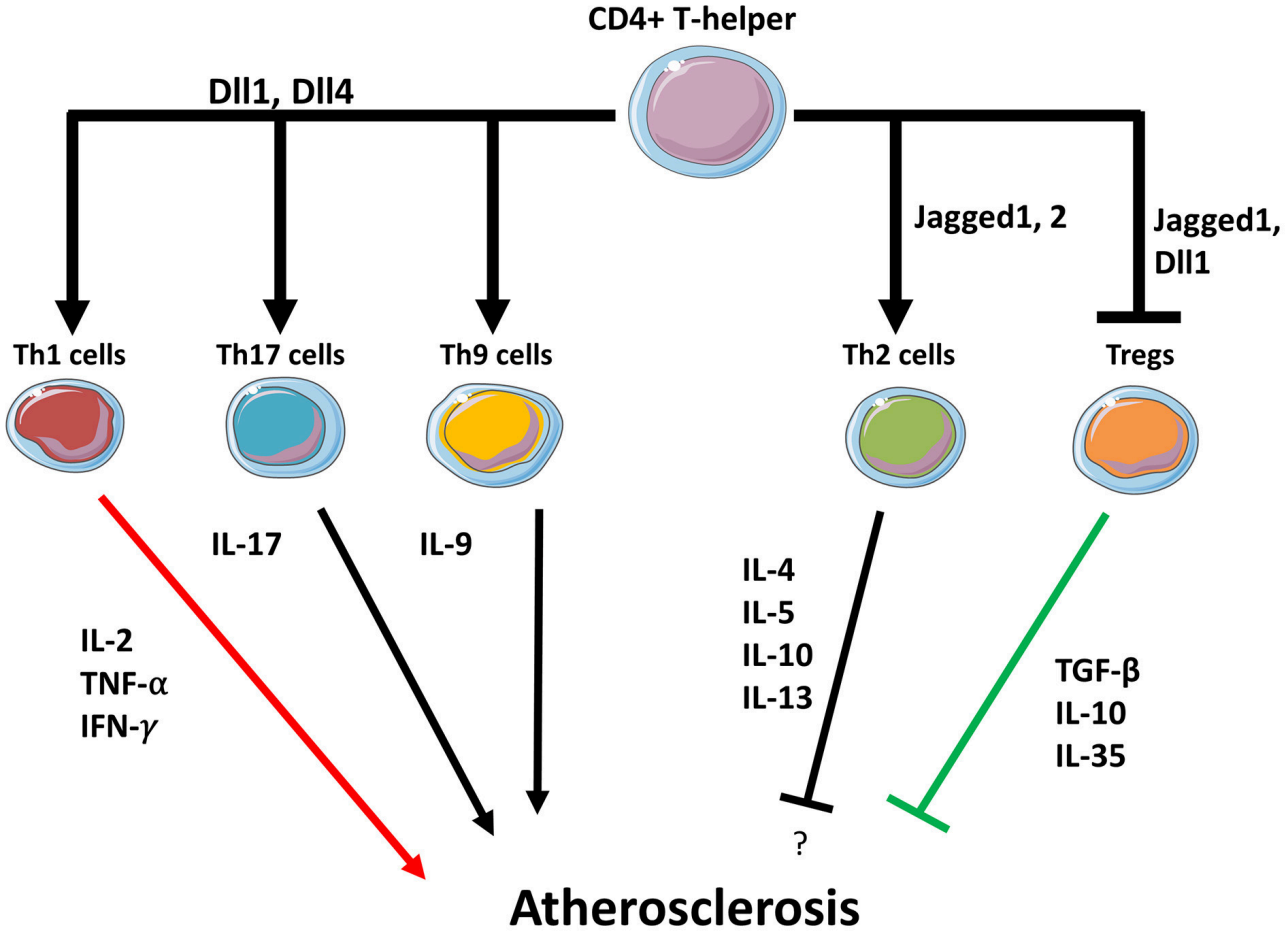
Notch may regulate T helper cells fate in the plaque. Dll1 and Dll4 ligands expressed in APCs promote Th1, Th9, and Th17 differentiation while suppressing Th2 differentiation. Jagged1 induces Th2 differentiation. Dll1 and Jagged1 both suppress Tregs activity.

Atherosclerosis is a chronic inflammatory disease driven by a complex interplay between vascular and immune cells, with an involvement of the Notch signaling pathway in each phase of the disease. Activation of Notch promotes atherosclerosis by inducing a pro-inflammatory M1 phenotype in macrophages at the expense of the M2 anti-inflammatory subtype. Even if the detailed molecular mechanisms are still not completely known, solid evidence has shown that the Dll4/Notch1 axis is pivotal in favoring M1 polarization, while blocking M2 immunosuppressive macrophages and their cytokines (Figure 1). T cells in atherosclerotic plaques can either promote–or protect from–the onset and progression of atherosclerosis. Notch ligands Dll1, Dll4, Jagged1, Jagged2 on APCS interact with Notch receptors on T-cells and this interaction regulate their differentiation. APCs expressing Dll1 or Dll4 promote the differentiation toward pro-atherosclerotic Th1 whereas Jagged ligands instruct T cells toward the less inflammatory Th2 subtype. Jagged also mediates the inhibitory activity of MDSCs on CD4 and CD8 T-cells (137). Notch signaling is also necessary for Tregs differentiation from naïve T cells, however, in already established Tregs Notch mediates the differentiation toward a Th1-like inflammatory phenotype (111). Although clear evidence demonstrates both the involvement of T cells in atherosclerosis and of Notch signaling in T cell regulation, the specific role played by the Notch signaling in T cells in the context of atherosclerosis has not been directly investigated. Based on the findings discussed in this review, it appears likely that Notch may favor atherosclerosis by promoting Th and CD8 cells formation (Figure 2). Consequently, inhibition of the Notch pathway could be a novel strategy to counteract inflammation of the vascular wall, and thus atherosclerosis, by interfering with the production of cytokines from M1 macrophages and with Th1 cells infiltration in the plaque. In principle, this strategy could have the advantage of increasing the immunomodulatory activity of M2 macrophages without depleting anti-inflammatory Tregs in the plaque.

Notch inhibition for cancer and other indications has been so far mainly relying on GSIs. GSIs are a heterogeneous group of small molecules that prevent Notch cleavage by the γ-secretase enzymatic complex, thus reducing the levels of active Notch (140). GSIs were developed based on their activity on Notch1 but can also inhibit, depending on the cell type, the cleavage of other Notch paralogs or interfere with other pathways (11, 13). Currently, there are several GSIs being investigated in Phase I and/or II clinical trials in cancer patients (clinicaltrials.gov). Specific drug administration approaches are required to limit their main toxic effect which is intestinal toxicity (141). Studies *in vivo* have shown that GSIs are able to interfere with the progression of atherosclerosis. Systemic administration of GSI (LY411,575 0.2, and 1.0 mg/kg/day for 8-weeks) suppressed Notch signaling in ApoE-deficient mice fed a high fat diet and reduced total plaque areas in the aortic sinus, plus macrophages from these mice showed reduced levels of ICAM-1 and migration ability. 0.2 mg/kg/day did not cause loss weight and alterations of intestine and thymus whereas with the 1 mg/kg/day dose intestinal and immunologic toxicity was observed, suggesting that only low doses of GSI could be used long term without adverse effects (45). Based on these results, GSI treatment in patients with atherosclerosis could dampen the inflammatory activities of macrophages; the overall effects on atherosclerosis progression would depend on how the treatment affects the complex interplay between acquired and innate immunity, and thus, the balance between pro- or anti-atherosclerotic T cells. For precise targeting of macrophages, mesoporous silica nanoparticles containing GSIs could be used to specifically deliver these molecules to these cells (142). Alternatively, cell-specific delivery to macrophages of Notch inhibitors miRNAs (143) using siRNA loaded exosomes could be another specific approach to block Notch and inflammation in these cells (49, 144). GSIs-coated stents could be used to prevent re-occlusion in some patients, after percutaneous intervention, since Notch could be also involved in restenosis due to its effect on promotion of vascular smooth muscle cells proliferation (145). Of interest, a recent study has shown that sulindac, a non-steroidal anti-inflammatory drug, interferes with triple negative breast cancer growth by inhibiting Notch in cancer stem cells without inhibiting Notch expression or cleavage in murine T cells (146). Based on these findings, it would be of interest to test the effect of this drug in the context of atherosclerosis, specifically in the regulation of Notch signaling in macrophages.

A clear understanding of the role played by each receptor and/or ligand in each cell of the innate and acquired immune system involved in atherosclerosis could lead to a more precise targeting of the Notch signaling by antibody–mediated blocking of a specific component of the pathway. Blocking antibodies against Dll4, Notch1, Notch2, or Notch3 are already being tested in phase I clinical trials in cancer patients (9). An anti-Dll4 antibody has been employed to successfully interfere with vascular inflammation and atherosclerosis progression in a mice model of atherosclerosis (48). Our data in peripheral artery disease (PAD) patients showing that intraplaque levels of Dll4 mRNA could be associated with the progression of the disease suggest that PAD patients could also benefit of this approach (147). Given the widespread expression of Dll4 in the vasculature and the immune system, concerns about toxicity of the treatment have been addressed. Long term administration (12 weeks) of agents blocking Dll4 in mice caused no toxicity in one study (48) whereas Yam et al. reported adverse effects in the liver (148). In cancer patients, administration of anti-Dll4 antibody caused heart failure in a subset of patients (149). Differently from Dll4, in the context of atherosclerosis Jagged1-mediated signaling could be protective, since anti-Jagged1 immunotherapy has been shown to inhibit MDSCs and overcome tumor-induced tolerance by activating T-cell (137). Consistently, high levels of Jagged1 mRNA intraplaque were associated to a less inflamed plaque profile and a slower progression of disease in PAD patients (147).

Clinical trials employing Tregs are ongoing in organ transplantation, type I diabetes, and graft vs. host disease. These trials mainly used naturally occurring FoxP3^+^Tregs from patients, followed by *in vitro* expansion and reinfusion. Of note, adoptive transfer of Tregs in mice models reduced atherosclerosis considerably, suggesting that a similar strategy may be beneficial in patients (150). On light of this, targeting of Notch pathway could be utilized to improve the generation and specificity of T-cells for adoptive transplant immunotherapies as already proposed for cancer-immunotherapy (151).

As discussed in previous paragraphs, the first step of atherosclerosis involves the interplay between the endothelium and infiltrating immune cells, step in which Notch1 in particular plays a crucial role by preventing the expression of adhesion molecules on ECs. Strategies aimed to prevent a reduction of Notch1 caused by turbulent shear stress (31) or dyslipidemia (25) or low estrogen conditions, as in post-menopausal women or breast cancer patients treated with anti-estrogens (152) could reduce endothelial dysfunction and therefore, plaque formation in atheroprone areas of the aortic endothelium. Heart rate reducing drugs (31), miRNA (30), or specific estrogen receptor agonist (24) could be used to prevent Notch1 downregulation in these areas.

The CANTOS study provided definitive prove that tackling inflammation by blocking IL-1β with the monoclonal antibody canakinumab can be a successful strategy to reduce atherosclerosis progression (2). It should be noted that canakinumab efficacy depends on the reduction of inflammation achieved. Individuals with hsCRP concentrations < 2 mg/L experienced a 25% reduction in cardiovascular events compared to a non-significant 5% reduction in those with on-treatment hsCRP levels ≥2 mg/L (153). Similarly, blocking IL-6 by a specific antibody has shown a protective effect in atherosclerosis even though the results of this treatment are still unclear (5). Targeting Notch in immune cells could represent a novel approach to counteract inflammation and thus atherosclerosis. To this aim a deeper knowledge of the specific roles of each Notch receptor and ligand in innate and adaptive immune cells, in the context of atherosclerosis, is needed.

## Author Contributions

FV, MV, and PR wrote the paper. GC, GA, and FF provided substantial revision. All authors reviewed and approved the final version of the manuscript.

### Conflict of Interest Statement

The authors declare that the research was conducted in the absence of any commercial or financial relationships that could be construed as a potential conflict of interest.
